# Telenutrition: Changes in Professional Practice and in the Nutritional Assessments of Italian Dietitian Nutritionists in the COVID-19 Era

**DOI:** 10.3390/nu14071359

**Published:** 2022-03-24

**Authors:** Patrizia Gnagnarella, Yvelise Ferro, Taira Monge, Ersilia Troiano, Tiziana Montalcini, Arturo Pujia, Elisa Mazza

**Affiliations:** 1 Associazione Tecnico Scientifica dell’Alimentazione Nutrizione e Dietetica (ASAND), Technical Scientific Association of Food, Nutrition and Dietetics, 90144 Palermo, Italy; patrizia.gnagnarella@ieo.it (P.G.); tmonge@cittadellasalute.to.it (T.M.); ersilia.troiano@gmail.com (E.T.); elisamazza@unicz.it (E.M.); 2 Division of Epidemiology and Biostatistics, IEO European Institute of Oncology IRCCS, 20141 Milan, Italy; 3 Department of Medical and Surgical Science, University Magna Grecia, 88100 Catanzaro, Italy; pujia@unicz.it; 4 Department of Clinical Nutrition, Molinette Hospital, 10126 Turin, Italy; 5 Direzione Socio-Educativa, Municipio Roma III Montesacro, 00137 Rome, Italy; 6 Department of Clinical and Experimental Medicine, University Magna Græcia, 88100 Catanzaro, Italy; tmontalcini@unicz.it

**Keywords:** telenutrition, nutrition assessment, COVID-19 pandemic, dietitians, telehealth

## Abstract

The COVID-19 pandemic has brought about various restrictions around the world, and its impact on healthcare has been enormous: RDNs have had to shift from in-person interactions with clients to telenutrition consultations, encountering obstacles. We designed the first survey to investigate the changes in RDN practices related to telenutrition provision after the onset of the pandemic through an online survey in Italy. Four hundred and thirty-six responses were analyzed. Before the pandemic, only 16% of Italian RDNs provided telenutrition; this percentage increased significantly up to 63% (*p* < 0.001). Among patients, the lack of interest in accessing telenutrition (30.9%) and the Internet (16.7%) were the most frequently reported barriers. Among RDNs, one of the main obstacles was their inability to conduct nutritional evaluation or monitoring activities (24.4%). Our survey indicated that increased adoption of telenutrition can be a valid, safe alternative to face-to-face visits. Telenutrition was mainly used by young RDNs (20–39 years) with fewer years of professional experience (0–20 years) and master’s degrees. Remote nutrition can enable RDNs to maintain normal workloads and provide patients with uninterrupted access to nutritional healthcare. It is important that RDNs using telemedicine resources possess the ability to provide high-quality, efficient, and secure services using evidence-based guidance.

## 1. Introduction

The global pandemic caused by COVID-19, a new coronavirus disease (SARS-CoV-2), brought about various restrictions around the world [1]. Many governments adopted restrictive measures regarding daily life, including social distancing, movement limitations, the closure of public services and schools, and forcing people to stay at home [1]. To control the viral spread and reduce the risks associated with COVID-19 in Italy, a lockdown was imposed starting from 9 March 2021 [2]. During the lockdown, in addition to schools, offices, stores, and most workplaces were closed, and only people in a state of absolute necessity were able to leave their homes [2]. Additionally, access to medical care was limited during the lockdown period to minimize the risk of transmitting SARS-CoV-2 [2].

The impact of the pandemic on healthcare has been huge in several respects. At the beginning of the pandemic, there was an emergency regarding beds for COVID-19 patients and the search for medical devices that would protect healthcare workers from infection. Subsequently, in an attempt to deal with the virus, other patient categories were affected. Hospital visits, outpatient activities by appointment, diagnostic screenings, and non-urgent surgeries were cancelled or postponed [3]. Some reports suggest that chronic patients have postponed seeking healthcare, some of them due to fears of contracting coronavirus infections in healthcare settings [4]. The PASSI d’Argento Italian survey showed that, among over 1200 persons interviewed, 44% declared that they had declined at least one medical examination (or diagnostic test) in the previous 12 months; 28% had to give one up due to the suspension of the service, while 16% did so voluntarily for fear of contagion [5]. The growing use of remote modalities for the management of diseases is certainly one of the elements that have characterized these two years of the pandemic. Telemedicine is “an innovative approach that allows the provision of remote health services through the use of digital devices, internet, software, and telecommunication networks” [6]. The digitalization of healthcare using telemedicine and new digital approaches has evolved in the last few years, and the unique circumstances of the COVID-19 pandemic resulted in its rapid and widespread implementation [7,8]. There are various benefits in using telehealth technology, especially in routine care and in cases where services do not require direct interaction with the patient [9,10].

During the COVID-19 pandemic, healthcare professionals faced professional and personal challenges [11]. Dieticians, as members of the healthcare team, play an important role in maintaining the wellness and health of individuals and communities, especially in the care of patients with chronic diseases and in caring for COVID-19 patients, determining their nutritional demands, and identifying risks of malnutrition or wasting during the hospital stay [11,12,13].

The Academy of Nutrition and Dietetics has recognized telenutrition as useful to implement in the context of the dietician’s activity and defined it as “The interactive use by an RDN of electronic information and telecommunications technologies to implement the Nutrition Care Process with patients or clients at a remote location, within the provisions of the RDN’s state license as applicable” [14,15]. RDNs have had to shift from in-person client interactions to telenutrition consultations—virtual consultations and remote video or audio technology-supported visits—to deliver nutritional assessments, menus, analysis, management plans, and follow-up to patients/clients [16].

There are currently few data on the use of telenutrition. A single US study showed that the number of RDNs who delivered telenutrition had increased considerably during the COVID-19 pandemic [17]. 

The main aim of this study was to investigate the conditions and changes in the work environment of Italian dieticians during the COVID-19 pandemic, with particular reference to the use of telenutrition. Furthermore, the other objectives were to evaluate the impact of the COVID-19 pandemic on clinical practice, investigate the strategies used to continue professional activity, and analyze the critical issues encountered by Italian RDNs.

## 2. Materials and Methods

The Technical Scientific Association of Food, Nutrition, and Dietetics (ASAND) designed an online investigation to measure dietitians’ nutrition activities during the COVID-19 pandemic in Italy. Between April and November 2021, after about a year from the beginning of the pandemic in Italy and after two phases of restrictions (9 March–18 May 2020, and 13 October 2020–25 February 2021), we conducted a national survey using an online survey link created using the Google Forms tool. The questionnaire was made available via online social media between 27 April and 30 November 2021. ASAND distributed the survey electronically by sending a link via email or newsletters, or posting the text and link on websites and social media (Facebook, LinkedIn, and WhatsApp) to facilitate the completion of the questionnaire by the RDNs living in Italy.

The aim was to reach the highest number of dietitians working in Italy, ASAND members (about 1000 Italian RDNs in 2021), and followers. The anonymity of the participants was guaranteed by the Google platform. Participation was voluntary, and non-monetary incentives were provided. All the individuals provided informed consent by agreeing to the data-protection declaration prior to starting the survey. The principles of the Declaration of Helsinki were followed, and the Local Ethics Committee approved the protocol in the Calabria Region—Central Area (205/2021/CE approved 20 May 2021).

### 2.1. Survey Development and Design

The survey questions (Supplementary Table S1) were developed by a group of experts of the ASAND executive committee. An adapted version of a survey previously published by the Academy [17] was produced. The survey comprised a total of 37 items (available at https://forms.gle/2GKZ3avP5n2DRFBf9 (accessed on 30 November 2021)). The questionnaire enquired about changes in RDNs’ practice related to the delivery of nutrition care via telemedicine after the onset of the COVID-19 pandemic [17]. It took respondents approximately 10 min to fill in. The questionnaire included sociodemographic questions, the highest academic qualification, the years of experience as a nutritionist, and experience providing nutrition care via telehealth before and during the COVID-19 pandemic. In addition, we investigated the current use of telemedicine, including critical issues in the management of patients through telenutrition.

### 2.2. Statistical Analysis

We enrolled a convenience sample. However, based on a previous published study [17], to detect variations in nutrition care via telehealth of at least 20% in RDNs, with 90% power with a two-sided level of significance of 0.05, a minimum of 128 individuals was needed. After closing the online survey and stopping data collection, the final database was downloaded as a Microsoft Excel sheet and the data were analyzed immediately thereafter.

The survey included some open and closed questions, and in some cases, multiple answers were expected. Only one survey was used for the analysis from participants who completed the survey multiple times (the first in chronological order). The respondents had the option to skip questions. Missing data were not imputed in the analysis.

The data are reported as means ± standard deviations (SDs) for continuous variables. Categorical variables are presented as absolute (n) and relative (%) frequencies. A Chi-square test was performed to analyze the change in the use of telemedicine before and during the COVID-19 pandemic. The participants were classified based on age groups, geographical provenance, and the highest degree earned. A Chi-square test was performed to analyze the proportion of the RDNs who provided nutritional care via telehealth during the COVID-19 pandemic. Significant differences were assumed to be present at *p* < 0.05 (two-tailed). All the comparisons were performed using SPSS 25.0 for Windows (IBM Corporation, New York, NY, USA).

## 3. Results

**Figure 1:**
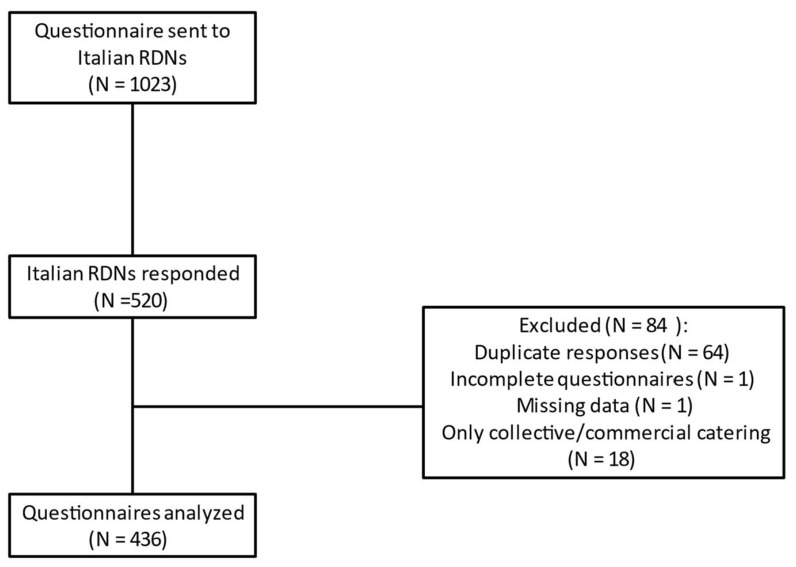
Flowchart of the study.

During the survey, a total of 520 responses were collected, of which only 436 (83.8%) were analyzed due to errors, missing data, or duplicate responses. In addition, the responses of dieticians who only dealt with collective or commercial catering were excluded (Figure 1).

The respondents’ mean age was 41 ± 12 years (range, 23 to 73). Of the participants, 99.5% (*n* = 436) were Italian registered dieticians and 63.3% (*n* = 276) were members of ASAND. The responding RDNs had a mean of 16 ± 11 years of experience in dietetics practice. Approximately 27% of the respondents had master’s degrees. The geographical provenance of the respondents was not homogeneous, with greater participation from dieticians living in Northern Italy (Northern = 57.1%, Central = 25.2%, and Southern = 17.7%); these data reflect the geographical distribution of ASAND associates. The main characteristics of the survey participants are summarized in Table 1.

**Figure 2:**
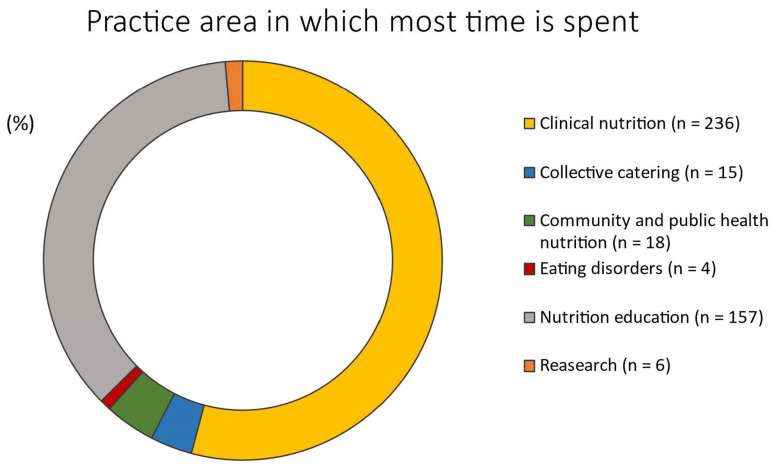
Practice area in which most time is spent by responders (*n* = 436).

The most frequently reported clinical areas were weight management (31.4%), diabetes care (11.5%), and disordered eating (11.7%) (Table 1). Although RDNs worked with individuals across a variety of age ranges, 87.6% of the RDNs reported working with adults between 22 and 64 years of age (Table 1). Clinical nutrition was reported as the primary practice area (Figure 2) by 54.1% of the responders, followed by 36% for nutrition education.

**Figure 3:**
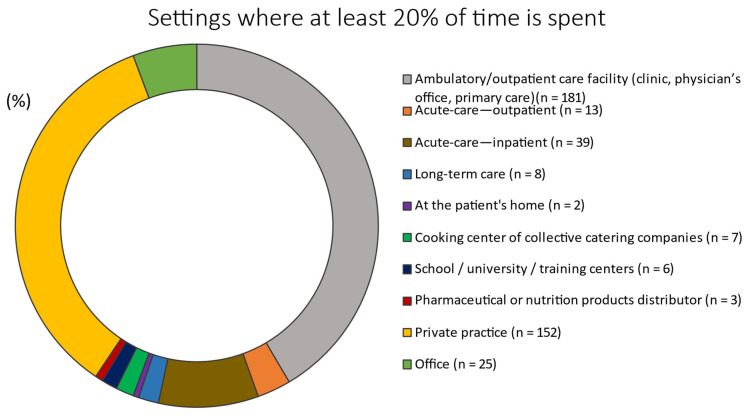
Setting where at least 20% of the time is spent (*n* = 436).

Approximately 41.5% and 34.9% of the RDNs spent at least 20% of their time in ambulatory/outpatient care facilities (e.g., clinics, physician’s offices, and primary care) or private practice, respectively (Figure 3).

**Figure 4:**
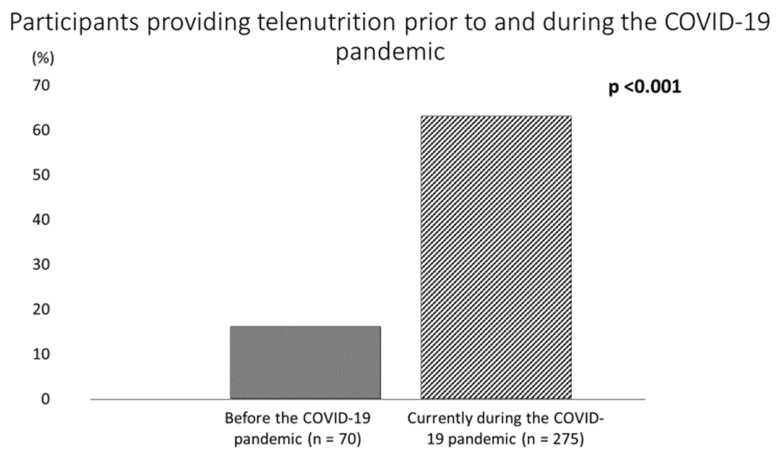
Participants providing telehealth prior to and during the COVID-19 pandemic.

Before the COVID-19 pandemic, Italian RDNs typically delivered face-to-face services for a mean of 22.3 ± 12 h per week (range, 1 to 55), and only 16.1% (*n* = 70) provided nutrition care via telehealth (Table 2 and Figure 4). However, this proportion increased significantly to 63.1% (*n* = 275) at the time the survey was completed (*p* < 0.001) (+293% increase) (Figure 4). The remaining 36.9% (*n* = 161) of the respondents did not use telenutrition during the pandemic.

Those who used telemedicine prior to the COVID-19 pandemic had a median of 4.7 ± 5 years of professional experience (Table 2). During the COVID-19 pandemic, the greatest proportion of RDNs (46.9%) reported exclusively using audio–visual modalities, while 32.4% reported using both audio–visual and telephone (audio-only) modalities to provide nutrition care via telemedicine (Table 2). Among the audio–visual options, the greatest proportion of RDNs used Google Meet (23.6%) to provide nutrition care via telehealth (Table 2). Telenutrition sessions are used primarily for individual meetings (78.5%) rather than groups (4.4%). Furthermore, RDNs indicated spending a mean of 47 ± 34 min per visit with patients per telehealth service (data not shown). The majority of the Italian RDNs reported assessing the following during the telehealth visits: nutrition and food consumption (84.7%), nutrition history (82.5%), and patients’ self-reported body measurements (62.9%) (Table 2). During the COVID-19 pandemic, 84% of the dieticians reported not changing the price of their nutrition care services (data not shown). Among the critical issues reported by patients were weight gain (33.1%), sedentary lifestyles (16%), emotional frailty, fear, anxiety, stress, depression (13.8%), and eating disorders (13.5%).

Table 3 describes some of the barriers and benefits reported by the RDNs during telenutrition. Among patients/clients, a lack of interest in accessing nutrition services via telemedicine (30.9%), and a lack of Internet access (16.7%) or a telephone (4%) were the most frequently reported barriers. Among the RDNs, a major barrier was their inability to properly conduct or evaluate nutrition assessments or monitoring activities (24.4%) (Table 3). Some RDNs (24%) indicated difficulties in establishing relationships and/or therapeutic alliances via telemedicine with their patients/clients. The benefits reported by RDNs were ensuring compliance with social-distancing guidelines for the COVID-19 pandemic (64.4%), improved patient/client access to nutrition services (44.4%), and scheduling flexibility (54.5%) (Table 3).

Lastly, we analyzed the demographic and professional characteristics of the RDNs who used telehealth to provide their nutritional care services during the COVID-19 pandemic. The RDNs were young adults (20–39 years) with fewer years of professional experience (0–20 years) and master’s degrees (Table 4). There was no statistically significant difference in the geographic provenance of the dieticians that currently used telemedicine (Table 4).

## 4. Discussion

This is the first survey investigating the conditions and changes in the work environment of Italian dieticians during the COVID-19 crisis, with particular reference to the use of telenutrition. Our survey described changes in RDN responsibilities and practices and discovered areas of need in telehealth compared with traditional care. Due to social-distancing guidelines, the number of RDNs delivering nutrition care services via telehealth significantly rose during the COVID-19 pandemic. Our investigation revealed that, prior to the COVID-19 pandemic, only 15% of the RDNs interviewed used telenutrition in Italy. During the pandemic, the use of telenutrition increased by nearly 300%, especially in private practice and ambulatory care settings [18,19]. Research conducted in the United States reported a 41% increase in the use of telemedicine among RDNs to provide healthcare compared with the pre-pandemic period (from 37% to 78%). However, several concerns were raised, such as a lack of best practices, health insurance coverage, billing, and reimbursement [17].

The RDNs who responded to our survey were freelancers with bachelor’s degrees and dealt mainly with bodyweight management. Moreover, our study showed that RDNs using telemedicine were young (less than age 40) with fewer years of professional experience and master’s degrees. This is partly due to the fact that young people are more likely to use new technologies [20]. Our results are in line with a survey conducted in other countries, where weight management was more common for freelance dietitians [17]. Numerous studies have shown that, during the COVID-19 lockdown, overweightness and obesity increased in all age groups [21,22,23]; therefore, the use of telenutrition for remote weight management was an excellent strategy for combating the increase in weight.

A randomized controlled trial showed that telenutrition interventions have improved weight-loss outcomes in cardiovascular-disease patients [24]. The same authors showed an improvement in weight statuses in obese patients via telenutrition [25]. Telemedicine proved useful in improving diabetes self-management in rural and ethnically diverse populations [26]. A systematic review reported that telenutrition improved diet quality and dietary adherence in patients with chronic diseases when compared with face-to-face dietary counselling [27].

The increased adoption of telenutrition, reported in our survey, indicates that it may be an acceptable alternative to traditional visits, even in the hospital setting. A recent systematic review [28] highlighted evidence that dietitians may improve nutrition care and patient, healthcare and/or workforce outcomes across the Nutrition Care Process (NCP) domains, particularly for patients with or at risk of malnutrition. It is well established that malnutrition and its associated complications negatively impact cost and clinical and patient-centered outcomes, including mortality [29]. Several studies have suggested that a significant proportion of patients with COVID-19 are at high risk of malnutrition [30,31]. Telenutrition could be the best way to ensure the continuity of the post-hospital nutritional care by RDNs to minimize the nutritional consequences of infection and optimize recovery at home. ASAND has made available guidelines for the nutritional assistance of SARS-CoV-2-positive, paucisymptomatic, or post-dismissal patients at home or in protected structures [32], to provide useful information for the evaluation and monitoring of nutritional status in compliance with the NCP that allows providing a safe and effective intervention.

A previous Italian survey found that telemedicine was managed on a largely voluntary basis in the pre-COVID-19 era without remuneration and without legal traceability for the offered services [33]. The COVID-19 pandemic has prompted a rapid restructuring of the healthcare system in an effort to stop the spread of the pandemic. Thus, telemedicine turned out to be preferable for healthcare professionals during the COVID-19 pandemic when face-to-face meetings were forbidden, allowing the provision of health services over a distance and the delivery of nutrition care services [14,15]. Healthcare professionals and dietitians have used telehealth techniques such as video conferencing and online consultations to support patients during lockdowns [11], helping clients by providing information and resources on lifestyle, food, and physical activity practices. The main modalities used by Italian RDNs to provide telenutrition were audio–visual modalities via Zoom/Google/Teams/Skype. Videoconferencing modalities for delivering nutrition care are less frequently utilized according to the published literature; however, they appear to be effective for managing diabetes and obesity. Some videoconferencing dietetic consultation research concluded that videoconferencing modalities could be attainable and well-accepted [34,35,36]. An Australian review [34] reported that videoconference-delivered nutrition care is as effective as face-to-face programs in terms of dietary outcomes. The same review compared in-person and videoconferencing nutrition care methods in people with diabetes and found clinical outcomes to be similar. All the studies analyzed in the review reported optimal patient satisfaction, improved diet adherence, and enhanced self-efficacy, with sufficient improvements in the biomarkers examined.

Face-to-face nutrition counseling is provided by RDNs or nutritionists, and involves tailoring or personalizing nutrition information to the patients’ requirements and lifestyle with the target of facilitating behavior change [37]. Face-to-face nutritional interventions are known to be effective in weight and dietary changes [38], and research has also shown that face-to-face nutrition care is important for feeling empowered, connected, and supported [39]. However, face-to-face nutrition counseling can be expensive and time consuming and may not be accessible to everyone [40]. The use of web-responsive applications, or telephone or audiovisual modalities provides an alternative to face-to-face nutrition care that can reach a greater number of individuals, especially in public health emergencies such as the COVID-19 pandemic. Recent evidence showed that web-based nutrition interventions may be successful in inducing short-term dietary change compared with standardized dietary interventions [37]. In addition, telenutrition makes it possible to participate in nutrition counseling in patients’ kitchens; this provides a window into their cooking and storage space and allows the counselor to review labels and products straight from the patient’s house. Telenutrition counseling was also appreciated by the patients for its flexibility, for saving travel time, and for the continuity of care, particularly for patients that lived in more remote areas. On the other hand, a recent study highlighted that, with telehealth, patients’ expectations are increased to 24-h availability [41]. In our survey, the RDNs reported spending approximately 45 min in direct contact with the individual/group per session, higher than the median of 30 min reported by Rozga et al. However, further studies are needed to investigate whether telenutrition can be used in place of traditional interventions for achieving body composition and dietary changes.

However, the RDNs reported difficulties in delivering some aspects of the nutrition care remotely, as clients were not interested in receiving nutrition services via telehealth (29%) or not able to properly conduct or evaluate some assessment or monitoring activities (28%) and establish relationships (17%) [17]. The data obtained from our study showed that the main obstacle to telenutrition is clients not being interested in receiving telenutrition, as well as difficulty with establishing relationships/therapeutic alliances via telehealth, or customers not having access to the Internet, as reported in a study conducted in the US [17]. Moreover, our results showed that our country is less technological than the US, the world’s most technologically advanced country. Consequently, Italian RDNs need further guidance on the best practices for conducting nutrition assessment and supporting successful service delivery via telehealth. In fact, dieticians’ work should be recognized since a lack of support and recognition might negatively affect their work performance [42], as reported by a recent study. It revealed poor compensation, a lack of professional recognition, and that dieticians’ well-being and quality of life decreased during the pandemic [42,43]. Moreover, healthcare professionals with low satisfaction and experiencing job stressors are more likely to provide poor-quality services and quit their jobs [18], as shown by US data [17]. Unfortunately, our survey is consistent with these results. In fact, 40% of the Italian RDNs who responded did not use telenutrition during the pandemic. This means that they had to suspend their work.

Our survey revealed important critical issues encountered in patients during telenutrition such as eating disorders, emotional frailty, fear, anxiety, stress, depression, and weight gain. Therefore, telenutrition could be an excellent method with which to closely monitor these emerging pandemic-related symptoms.

Our study shows how telenutrition, like other branches of telemedicine, improves access to care, reduces the use of resources in health centers, minimizes the risk of the transmission of infectious agents [32], and provides wide access to caregivers [44]. Therefore, this technology is an effective, attractive, and affordable option [45] in this period and beyond.

Our study highlights how it is necessary for Italian RDNs to have univocal guidelines to follow in order to implement telenutrition. It is also important to carry out awareness campaigns for clients who approach nutrition assessment face to face to increase the use of telenutrition, especially in this pandemic period. In addition, our study highlights how the adaptation of local systems with changes regarding payment and the coordination of services are barriers to the large-scale use of telenutrition to deal with the COVID-19 pandemic.

This study should be interpreted with caution given some limitations and bias mainly due to its nature (survey, self-reported, and online), but an online survey is a recommended approach for quickly reaching a specific group of individuals and guaranteeing their safety under a pandemic [24,46]. Other limitations are related to the use of modified questionnaires, but we paid particular attention to reaching the highest number of RDNs and facilitating the compilation of the questionnaire. Still, the topic might have selected responders (self-selection bias) who previously accessed telenutrition. The youngest dietitians are more likely to use the Internet and social media and can partially explain the low response rate. For this reason, it is difficult to determine the generalizability of the results for the greater RDN population. Finally, we cannot rule out the possibility that RDNs could be in deep distress, as these individuals might have either been on leave due to their concerns about the pandemic or extremely busy working in the intensive care unit and/or surveillance unit, and, consequently, unable to join the study.

## 5. Conclusions

This study is the first survey to evaluate the impact of the COVID-19 pandemic on RDN practices in Italy. Telenutrition can enable RDNs to maintain normal workloads and provide patients with uninterrupted access to nutritional health care. While it is too soon to know the long-term impacts of the COVID-19 pandemic on the Italian healthcare delivery system, our results provide a picture of how telehealth can enhance nutrition care. Further studies should be planned to monitor successes and obstacles for RDNs offering telehealth during and after the COVID-19 health crisis. It is important that RDNs utilizing telehealth resources possess the capacity to provide high-quality, efficient, and secure services using evidence-based guidance.

## Figures and Tables

**Table 1:** Description of the participants’ demographic and professional characteristics (*n* = 436).

Characteristics	*n*	%
Age groups (years)		
20–39	228	52.3
40–59	167	38.3
60–75	41	9.4
Geographical provenance		
Northern Italy	249	57.1
Central Italy	110	25.2
Southern Italy	77	17.7
Highest degree earned		
Bachelor’s Degree	233	53.4
Master’s Degree	116	26.6
1st level University Master’s Degree	69	15.8
2nd level University Master’s Degree	11	2.5
Academic Doctorate Degree	7	1.6
Member of ASAND °		
Yes	276	63.3
No	160	36.7
Current work ^ϭ^		
NHS employed	146	33.5
Private healthcare facility employee	27	6.2
Freelance	243	55.7
Employed by two institutes/centers ^#^	12	2.8
University professor	2	0.5
Other	6	1.3
Experience as dietitian (years)		
0–10	185	42.4
11–20	123	28.2
21–30	75	17.2
31–40	41	9.4
41–50	12	2.8
Focus area in which most time is spent		
Artificial nutrition	16	3.7
Diabetes care	50	11.5
Disordered eating	51	11.7
Food and nutrition consultant	44	10.1
Food manager in collective catering companies	17	3.9
Gastroenterological support	18	4.1
Gerontological nutrition	10	2.3
Health prevention and nutrition education	9	2,1
Oncology	20	4.6
Other ^§^	13	3.0
Kidney disease nutrition	14	3.2
Sports nutrition	17	3.9
Weight management	137	31.4
Women and pediatric nutrition	20	4.6
Age range of studying populations *		
Older adults (age 65+)	187	42.9
Adults (ages 22–64)	382	87.6
Pregnant/postpartum women	122	28
Teenagers and young adults (ages 13–21)	182	41.7
Children (ages 6–12)	105	24.1
Young children (ages 1–5)	44	10.1
Infants	17	3.9

° ASAND = Technical Scientific Association of Food, Nutrition and Dietetics. ^ϭ^ NHS = National Health System; ^#^ Employed by two institutes/centers = dietician who works simultaneously for the NHS or private healthcare facility and for themselves; Other = PhD students or job contract. ^§^ Other: bariatrics; cardiovascular; neurology; hereditary metabolic diseases; autoimmune diseases; university research; clinical studies. * Participants were able to select all options that applied.

**Table 2:** Participants’ professional experiences providing telenutrition prior to and during the COVID-19 pandemic.

Prior to COVID-19 Pandemic	Mean ± SD	
Hours per week providing face-to-face nutrition care (*n* = 417) ^≠^	22.3 ± 12	
Years of experience providing nutrition care via telehealth (*n* = 70)	4.7 ± 5	
During the COVID-19 pandemic	*n*	%
Targets of patients via telenutrition		
Individuals	216	78.5
Groups	12	4.4
Both individuals and groups	47	17.1
Current modalities used to provide telenutrition		
Telephone (audio only)	47	17.1
Audiovisual	129	46.9
Both telephone and audiovisual	89	32.4
Other ^§^	10	3.6
Audiovisual options used to provide telenutrition		
Audiovisual capability built into the electronic health record	7	2.5
Google Meet	65	23.6
Lifesize	6	2.2
Teams/Cisco WebEx Meetings/WebEx Teams	23	8.4
Zoom	5	1.8
Zoom/Google/Teams/Skype	77	28.0
WhatsApp	23	8.4
WhatsApp, Skype	12	4.4
Healthcare specialized platforms	14	5.1
Other ^#^	43	15.6
Types of nutrition assessment and/or monitoring and evaluation conducted via telehealth *		
Self-reported body measurements	173	62.9
Food and nutrition assessment	233	84.7
Evaluation of knowledge/beliefs/attitudes	184	66.9
Nutritional history	227	82.5
Behaviors	34	12.4
Assessment/monitoring tools	30	10.9
Physical activity and function	171	62.2
Biochemical data	11	4.0
Types of nutrition interventions provided via telehealth *		
Coordination of nutrition care	30	10.9
Nutrition counseling	220	80.0
Nutrition education	215	78.2
Nutrition prescription	109	39.6
Nutrition supplementation	39	14.2
Enteral and parenteral nutrition	27	9.8
Groups of population-based nutrition action	44	16.0
No intervention	10	3.6
Critical issues encountered in patients during telenutrition *		
Unhealthy eating habits	10	3.6
Eating disorders	37	13.5
Obstacles to care access	5	1.8
Emotional eating	2	0.7
Emotional frailty, fear, anxiety, stress, depression	38	13.8
Weight gain	91	33.1
Malnutrition	5	1.8
Redaction of economic possibilities	8	2.9
Poor compliance	17	6.2
Sedentary lifestyle	44	16.0
None	16	5.8

^≠^ RDNs (*n* = 19) reported zero hours per week providing face-to-face nutrition care because they were already doing telenutrition_._^§^ Other: telephone and email; ^#^ Other: healthcare-specialized platforms. * Participants (*n* = 275) were able to select all options that applied.

**Table 3:** Barriers and benefits encountered by RDNs providing telenutrition during the COVID-19 pandemic.

Barriers to Providing Telenutrition *	*n*	%
Not being able to conduct or evaluate some typical assessment or monitoring/evaluation activities	67	24.4
Not being able to deliver some routine nutrition interventions	24	8.7
Not having equipment to deliver telenutrition at home	11	4.0
Not having remote access to the electronic health record at home	16	5.8
Clients not having a telephone (landline or mobile phone)	11	4.0
Clients not having access to the Internet	46	16.7
Clients not interested in receiving telenutrition	85	30.9
Payer(s) do not include RDNs in their provider networks	12	4.4
Payer(s) do not include nutrition services in their telehealth policies	21	7.6
Lack of employer support	12	4.4
Difficulty of establishing relationships/therapeutic alliance via telehealth	66	24.0
Discomfort with delivering nutrition care via telehealth	24	8.7
None	67	24.4
Benefits experienced by delivering telenutrition *		
Improved patient access	122	44.4
Scheduling flexibility	150	54.5
Reduced transportation costs for patients/clients	117	42.5
Promoting compliance with social distancing measures recommended due to COVID-19 pandemic	177	64.4
None	5	1.8

* Participants (*n* = 275) were able to select all options that applied.

**Table 4:** Description of the demographic and professional characteristics of the RDNs providing telehealth during the COVID-19 pandemic.

	Age Groups			*p*-value
	20–39 yrs (*n* = 228)	40–59 yrs (*n* = 167)	60–75 yrs (*n* = 41)	
Providing telenutrition (%)	70	56	56	0.007
	Geographical provenance			
	Northern Italy (*n* = 249)	Central Italy (*n* = 110)	Southern Italy (*n* = 77)	*p*-value
Providing telenutrition (%)	65	58	64	0.56
		Degree earned		*p*-value
	Bachelor’s Degree/1st Level University Master’s Degree (*n* = 302)	Master’s Degree (*n* = 116)	2nd Level University Master’s Degree/Academic Doctorate Degree (*n* = 18)	
Providing telenutrition (%)	59	72	72	0.019
	Experience as RDN			*p*-value
	0–20 yrs (*n* = 308)	21–40 yrs (*n* = 116)	41–50 yrs (*n* = 12)	
Providing telenutrition (%)	66	59	25	0.005

## Data Availability

The data presented in this study are available on request from the corresponding author.

## Supplementary Materials

The following supporting information can be downloaded at: https://www.mdpi.com/article/10.3390/nu14071359/s1, Table S1: Changes in the professional practice of the Italian Dietitian following the COVID 19 pandemic.
