# Supplementary material for: Telenutrition: Changes in Professional Practice and in the Nutritional Assessments of Italian Dietitian Nutritionists in the COVID-19 Era
Source: Nutrients. 2022 Mar 24;14(7):1359. doi: 10.3390/nu14071359 (PMC9002661; doi:10.3390/nu14071359)
Supplement: Supplementary file 1 [file nutrients-14-01359-s001.zip › nutrients-1629367-supplementary.pdf]

SUPPLEMENTARY TABLE 1.

Changes in the professional practice of the Italian Dietitian following the COVID 19 pandemic
Dear Colleague,<br>We are conducting a survey to investigate the changes in the professional practice of Italian dieticians due to the containment measures and epidemiological management of Covid-19.<br>This is a research promoted by ASAND - TECHNICAL SCIENTIFIC ASSOCIATION OF FOOD, NUTRITION AND DIETETICS.<br>The compilation of this questionnaire is anonymous and takes a few minutes. Please click on the answer that you consider closest to your opinion.<br>The answers will remain confidential and will be used exclusively for statistical purposes in compliance with current legislation on the confidentiality of personal data.<br>The data collected will be processed in compliance with the privacy legislation (Information for the processing of personal data, pursuant to Legislative Decree 196/2003 coordinated with Legislative Decree 101/2018).<br>Thanks for the precious collaboration.
e-mail
**Are you registered in the Register of Dietitians?**
Yes
No
**Are you registered in ASAND?**
Yes
No
**How old are you?**
Please enter the number of years
**In which Italian region do you work as a Dietitian?**

Please enter the regions where you work
**What is the major university degree you have obtained?**
university degree / three-year degree or equivalent
1 st level master
specialist / master's degree
Second level master
Ph.D
**How many years have you been working as a Dietitian?**
Please enter the number of years
**How many hours a week did you spend on face-to-face nutritional care before the Covid-19 pandemic?**
Please enter the number of hours
What is your current position?
national health system employee
employee of a private facility
freelance
contractor / occasional collaboration
Other
**Which area of nutrition do you devote most of your time (>= 50%) to in your professional practice?**
clinical nutrition
community and nutrition in public health
nutritional education

Collective catering
Research
industry
sport
Other
**Where do you spend at least 20% of your time in the course of your profession?**
clinic / outpatient facility (e.g. clinic, doctor's office, primary care, etc.)
acute / outpatient care service
Long Term Care facility
private practice
acute care / hospital service
office
cooking center
Other
**What area of professional interest do you spend the most time on? (> = 50%)**
nephrology
diabetes
elderly
body weight management
eating disorders
oncology
Gastroenterology
artificial nutrition
pediatric
consulting
management of meals and menus in collective catering

sporty
Other
**What is the age range of the subjects to which you offer your professionalism?**
seniors (+ 65 years old)
adults (22-64 years)
adolescents and young people (13-21 years)
school age children (6-12 years)
toodler (1-5 years)
newborns
pregnant / postpartum women
**Did you use social media in your professional practice before the pandemic?**
Yes
No
**If so, which social networks? list social media or write "no"**
**Has the use of social media in your professional practice changed with the pandemic?**
Yes
No
**If so, what social media are you using? list social media or write "no"**
**Have you ever used telemedicine before the COVID-19 pandemic?**
Yes
No

**How many years did you provide nutritional care via Telemedicine before the COVID-19 pandemic?**
Please enter the number of years
**Do you currently provide nutritional care via telemedicine?**
Yes
No
**Who do you currently offer your telemedicine consultations to?**
individuals
groups
both individuals and groups
nobody
**What methods do you currently use for your telemedicine consultations?**
telephone (voice only)
audio-Video
both
nothing
Other···
**What audio-video options do you use to carry out your telemedicine consultations?**
Zoom
audio-video functions integrated in the electronic medical record
Teams / Cisco Webex Meetings / Webex Teams
Meet
nothing
Other···

**How many minutes do you usually spend on telemedicine counseling?**
Please enter the number of minutes
**What nutritional assessment and / or assessment technique do you use via Telemedicine?**
dietary and nutritional survey
knowledge / beliefs / attitudes
nutritional history
behaviors
evaluation / monitoring tools
physical activity and physical function
factors affecting access to food and food supplies
biochemical data, medical tests and procedures
use of drugs and complementary / alternative medicine
anthropometric measurements
administration of food and nutrients
physical results focused on nutrition
nothing
Other
**What nutritional interventions do you provide through Telemedicine?**
nutritional advice
food education
nutritional prescription
integrative dietary therapy
coordination of nutritional assistance by a nutrition professional
enteral and parenteral nutrition
nutritional aspects aimed at groups

none
Other···
**What methods do you use to communicate the results obtained with telemedicine to its managers?**
I document the interaction through an electronic medical record
I inform the service manager by e-mail
I inform manager by fax
I inform the service manager by telephone
I don't communicate anything about my interaction
I don't use telemedicine
Other
**Is billing done for your telemedicine consultations?**
Yes, always
Yes, sometimes
no
I do not know
I don't do telemedicine consultations
**How has your number of patients/clients evolved since the start of the pandemic?**
Increased
the same
Reduced
I do not know
I don't do nutritional counseling
**Has the request of your patients/clients changed since the start of the pandemic?**

Yes
No
I don't do nutritional counseling
**What are the main issues you have encountered in your patients/clients as an effect of the pandemic?**<br>**please list the problems encountered**
**Have you changed the price of your consultancy / services since the start of the pandemic?**
yes
no
I do not carry out any advice / services
**Did you encounter obstacles in carrying out your professional practice through telemedicine?**
customers not interested in receiving nutritional services via telemedicine
not being able to conduct or evaluate some typical evaluation or monitoring / evaluation activities
customers who do not have access to the Internet
customers not interested in receiving nutritional services at this time
difficulty in establishing therapeutic relationships / alliances through telemedicine
not being able to provide some routine nutritional interventions
discomfort in providing nutritional care via telemedicine
health facilities / services do not include nutritional services in their telemedicine policies
customers without telephone (landline or mobile)
not have remote access to the electronic medical record at home
having no equipment to deliver telemedicine at home
the paid services do not include the dietitian's consult
lack of support from the employer

not have access to my institution's planning system at home
I do not carry out any consult / services
no
other
**What benefits have you experienced from telemedicine?**
promote compliance with recommended social distancing measures due to the COVID-19 pandemic
greater flexibility in planning
reduction of transport costs for clients
easier access to care for patient
I do not carry out any advice / services
Other
**Have you consulted any specific guidance on how to provide nutritional care through telemedicine during the COVID-19 pandemic?**
yes
no
I'm not sure
I do not carry out any consult / services
**What is its main source of updating?**
employer
professional register
Association of Nutrition and Dietetics of Dietitians-ASAND
other associations
nobody
Other…

**Was the material produced by ANDID / ASAND during the pandemic useful?**
Yes
No
I do not know it
**What support would you like to receive in this period from your Association?**
Please insert your comment
